# Implication of Interleukin Family in Cancer Pathogenesis and Treatment

**DOI:** 10.3390/cancers13051016

**Published:** 2021-03-01

**Authors:** Manuela Gonzalez-Aparicio, Carlos Alfaro

**Affiliations:** 1 Gene Therapy Program, Fundación para la Investigación Medica Aplicada, CIMA, Universidad de Navarra, Instituto de Investigación Sanitaria de Navarra (IdiSNA), Av. Pio XII 55, 31008 Pamplona, Spain; gamanuela@unav.es; 2 Centre for Applied Medical Research, Universidad de Navarra, Av. Pio XII 55, 31008 Pamplona, Spain

## 1. Cytokines and their Side Effects

Cytokines are small proteins that are crucial for controlling the growth and activity of blood cells and other cells of the immune system. When released, they have the ability to send a signal to the immune system to fulfill its specific function. Cytokines affect the growth of all blood cells and other cells that help immune and inflammatory responses. There are different types of cytokines, including interleukins, interferons, tumor necrosis factors, and growth factors. Actually, some cytokines can be made in a laboratory and are used to treat cancer. The most common are interleukins and interferons.

## 2. Interleukins

Interleukins are a group of cytokines that act as chemical signals between white blood cells. It is a very large family and each of them has a specific activity and has a specific function. Several of them are highly represented in the organism and are metabolically more important in their direct implication in tumor pathogenesis. Two possible scenarios must be distinguished: (1) the use of interleukins directly as an anti-tumor treatment via blocking or increasing interleukins with different immunological strategies, or (2) the use of interleukins as a biomarker/indicator of tumor remission or progression.

## 3. Special Issue “Interleukin in Cancer Pathogenesis and Treatment”

In this Special Issue, we have compiled a series of 10 articles (four original articles, five reviews and one commentary) presented by international leaders in cancer immunology and new cell therapies.

We start this extensive list with IL-1. It is widely described that IL-1 is produced and secreted by various cell types, such as immune cells, fibroblasts, or cancer cells. Additionally, in cancer IL-1 has pleiotropic effects on immune cells, angiogenesis, cancer cell proliferation, migration, and metastasis. For this reason, the groups of Ghiringhelli and Kobold [1,2] present a clear overview of past and present research focusing on the role of IL-1 in cancer, with a special interest on clinical research and on therapeutic implications.

The next interleukins we consider are IL-2 and IL-15. Lundqvist et al. [3] discuss the distinct roles of both interleukins in activating certain functions of immune cells, with a particular approach on the signals implicated in the resistance of immune suppressive factors related to the tumor microenvironment. The review summarizes modifications of these cytokines to amplify their antitumor efficacy while minimizing toxicity and the clinical applications in metastatic cancer.

The implications of IL-4 and IL-13 in cancer are shown by the group of Krzystek-Korpacka [4]. Both immunosuppressive interleukins may directly promote cancer pathogenesis, but neither their status nor their role in the gastrointestinal tract is still clearly clarified. The article exposes that certain gastrointestinal tract cancers are associated with IL-4 and IL-13 upregulation, which may facilitate cancer growth. In conclusion, the authors suggest that targeting both interleukins as an antineoplastic strategy warrants further investigation.

Some of the most important interleukins in inflammatory processes are IL-6 and IL-10. In this edition, two different articles deal with both interleukins [5,6]. One study evidences the use of IL-6 and lymphocyte count as a possible biomarker to guide clinical decisions on prostate cancer treatment based on a multidisciplinary approach [5]. By way of confirming the impact of IL-6 and IL-10 polymorphisms on the prognosis of patients with diffuse large B-cell lymphoma (DLBCL), the group of Kewan [6] showed no significant differences in the distribution of all IL-10 polymorphisms studied of DLBCL between patients and controls. Likewise, the IL-6 rs1800797 was the only single nucleotide polymorphism (SNP) to show significant survival results in this study.

On the other hand, it is important to mention IL-8, another proinflammatory interleukin reviewed by our group [7]. Levels of IL-8, which can act upon a variety of immune and nonimmune cells, can tell us a lot about tumors, including their size (positive association) and how likely they are to respond to immunotherapy (negative association). Currently, the field that relates IL-8 to immunotherapy is leading to numerous and promising clinical trials that combine the inhibition of IL-8 with existing immunotherapeutic therapies and also its direct application as a promising tumor biomarker.

It is necessary to mention the implication of IL-12 in cancer treatment. Our group tested the efficacy of immunotherapies using cytokine genes or co-stimulatory molecules [8]. IL-12 is a potent immunostimulatory cytokine, which has shown properties as an anticancer agent in experimental tumors, as a recombinant protein or in gene therapy approaches, as has been proven in numerous clinical trials.

Continuing with the progression, IL18 has also been examined. The group of Luft [9] provided a rationale to explore the modulation of interleukin-18 for improving hematopoietic recovery and outcomes in allogeneic stem cell transplantation recipients.

Lastly, in this Special Issue, the implication of IL-34 in cancer has been reviewed [10]. The Monteleone group has raised a great review about the multiple roles of IL-34 in various cancers. They clearly state the relationship between the expression of this cytokine and cancer behavior, and provide new insights for exploring a new potential therapeutic target.

Finally, a review by Allegra et al. and an interesting commentary by Krause et al. [11,12] provide clear evidence examining the role played by cytokines in B-cell lymphocytic leukemia. Modifications in the cytokine balance could support the growth of the leukemic clones via the modulation of cellular proliferation and apoptosis. It is a fact that cytokines play pivotal roles in the pathogenesis of B cell malignancies provided by both in vitro data and clinical trials in lymphocytic leukemia patients.

In summary, this Special Issue of *Cancers* is a collection of articles discussing the role of interleukins in tumor development, with a special reference to cancer pathogenesis and possible treatments whose target is mainly the modulation of these proteins, as double-edged swords that we are beginning to know.
